# Bax Function in the Absence of Mitochondria in the Primitive Protozoan *Giardia lamblia*

**DOI:** 10.1371/journal.pone.0000488

**Published:** 2007-05-30

**Authors:** Adrian B. Hehl, Attila Regos, Elisabeth Schraner, André Schneider

**Affiliations:** 1 Institute of Parasitology, University of Zürich, Zurich, Switzerland; 2 Institute of Veterinary Anatomy, University of Zürich, Zurich, Switzerland; 3 Department of Cell and Developmental Biology, University of Fribourg, Fribourg, Switzerland; Newcastle University, United Kingdom

## Abstract

Bax-induced permeabilization of the mitochondrial outer membrane and release of cytochrome c are key events in apoptosis. Although Bax can compromise mitochondria in primitive unicellular organisms that lack a classical apoptotic machinery, it is still unclear if Bax alone is sufficient for this, or whether additional mitochondrial components are required. The protozoan parasite *Giardia lamblia* is one of the earliest branching eukaryotes and harbors highly degenerated mitochondrial remnant organelles (mitosomes) that lack a genome. Here we tested whether human Bax expressed in *Giardia* can be used to ablate mitosomes. We demonstrate that these organelles are neither targeted, nor compromised, by Bax. However, specialized compartments of the regulated secretory pathway are completely ablated by Bax. As a consequence, maturing cyst wall proteins that are sorted into these organelles are released into the cytoplasm, causing a developmental arrest and cell death. Interestingly, this ectopic cargo release is dependent on the carboxy-terminal 22 amino acids of Bax, and can be prevented by the Bax-inhibiting peptide Ku70. A C-terminally truncated Bax variant still localizes to secretory organelles, but is unable to permeabilize these membranes, uncoupling membrane targeting and cargo release. Even though mitosomes are too diverged to be recognized by Bax, off-target membrane permeabilization appears to be conserved and leads to cell death completely independently of mitochondria.

## Introduction

The unicellular intestinal parasite *Giardia lamblia* (syn. *G. intestinalis, G. duodenalis*) is a major cause of diarrheal disease in humans and animals worldwide [1]. *Giardia* belongs to one of the earliest branches of the eukaryotic lineage known to date [2], [3] and does not have a classic apoptotic pathway; both, caspases and Bcl-2-like proteins are not present. Furthermore, *Giardia* lacks bona fide mitochondria involved in oxidative phosphorylation [1]. Recently, mitosomes, i.e. putative vestigial organelles with mitochondrial origins, have been identified and characterized [4]. Mitosomes are small, double membrane-bounded organelles devoid of DNA, which function in the maturation of iron-sulphur proteins [5], [6], but are not involved in energy metabolism. Giardial mitosomes lack a genome, but genes of mitochondrial origin have been found in the nuclear DNA [4]–[8]. All available data support the hypothesis that mitosomes are vestigial organelles whose origins date back to the single endosymbiontic event leading to the establishment of mitochondria in the eukaryotic lineage. Mitosomes have been massively reduced in size and lost their energy-generating functions during the long independent evolution of the diplomonads [4]–[9], presumably as a consequence of an ancestor adapting to microaerophilic niches. Morphologically, the organelles can be divided into two distinct populations: the small, spherical peripheral mitosomes (Pm) distributed randomly in the cytoplasm, and the elongated central mitosome (Cm) whose localization is fixed at the basal body complex in the cell center [6]. Only the Cm is divided during mitosis and actively partitioned to daughter cells. How these organelles arise and how their constant size and number is maintained is unknown, however [6].

Synthesis and secretion of a protective extracellular matrix (cyst wall) as well as accompanying morphological changes are essential for survival of infectious stages of *Giardia* in the environment and thus for transmission [1]. Encystation in vitro or in vivo is triggered by environmental cues, for example cholesterol concentrations in the gut contents. The secreted cyst wall material consists of at least three paralogous cyst wall proteins (CWP1–3) [10]–[12] between 26 and 39 kDa and β(1–3) GalNAc homopolymers [13]. CWPs are synthesized in the endoplasmic reticulum (ER) and concentrated in large developmentally regulated Golgi-like compartments termed encystation-specific vesicles (ESVs), where they undergo processing and oligomerization before being secreted. ESVs arise apparently de novo and contain only pre-sorted cargo destined for the cyst wall [14]–[16]. Taken together with their ability to recruit Golgi marker proteins and their sensitivity to brefeldin A, ESVs are possibly an evolutionary early version of Golgi cisternae, which are functionally restricted to post translational maturation of proteins [17].

Members of the Bcl-2 protein family tightly control the mitochondrial pathway of apoptosis in higher eukaryotes and can be divided into pro- and anti-apoptotic members. During apoptosis, the proapoptotic Bcl-2 family member Bax translocates from the cytosol to mitochondria and causes activation of the caspase cascade by releasing cytochrome c from the intermembrane space [18], [19]. The signal triggering Bax activation has not been identified yet. Nevertheless, over-expression of Bax in mammalian cells was sufficient for mitochondrial targeting and the release of cytochrome c [20]. Furthermore, it has been shown that human Bax targets mitochondria and leads to cell death in yeast [21] and trypanosomes [22], and expression of myc-tagged Bax caused cytoplasmic release of cytochrome c in yeast, similar to the effect observed in mammalian cells [23]. Although Bax acts specifically on mitochondria in vivo, Bax alone is capable of pore formation in artificial lipid bilayers exposed to recombinant Bax protein, as revealed by atomic force microscopy of [24]. However, whether in vivo Bax alone is sufficient to induce cytochrome c release or whether it needs additional mitochondrial components is a matter of debate [25], [26].

Because it is unclear whether *Giardia* mitosomes harbor essential functions for the parasite or whether they are sufficiently reduced to be dispensable, we conditionally expressed human Bax in *Giardia* in an attempt to compromise these organelles. This strategy is based on the assumption that the organelles would be sufficiently conserved to allow specific recognition of heterologous Bax protein. However, we found that Bax does not physically interact with or affect mitosomes, which is suggestive of a high degree of degeneration. Instead, a serendipitous observation showed that in cells undergoing stage-differentiation, ESV organelles were damaged and lost their cargo proteins. Here, we analyze this unusual membrane interaction which demonstrates for the first time in a living organism the general ability of the Bax protein to target and compromise compartment membranes in the absence of additional mitochondria-specific factors.

## Materials and Methods

### *Giardia* Culture, Transfection and Analysis of Transgenic Cells

*Giardia lamblia* strain WBC6 (ATCC catalog No. 50803) trophozoites were grown in TYI-S-33 supplemented with 10% fetal bovine serum and bovine bile. The two step encystation method was used to induce expression of genes under the control of the CWP1 promoter as described previously [27], by increasing the medium pH and by addition of porcine bile after culture for ∼44 h in medium without bile. For Bax inhibition studies the peptide inhibitor Ku70 (Calbiochem, San Diego, CA) was added together with the encystation medium at a final concentration of 200 µM.

Plasmid DNA containing genes of interest and selectable markers was electroporated into trophozoites, and stable transgenic cells were selected using the antibiotic G418 (Sigma, St. Louis, MO), as described previously [27]. Plasmids were maintained episomally under continuous antibiotic selection.

All expression constructs were based on the cassette C1-CWP for expression under the control of an inducible CWP1 promoter [27]. The full-length or C-terminally truncated human Bax ORF was amplified by PCR from a plasmid [22] using primers (5′ to 3′ orientation) ATGCATGACGGGTCCGGGGAG (sense ), TTAATTAATCAGCCCATCTTCTTCCAGAT (full-length antisense), CCTTAATTAATCACCACGTGGGCGTCCCAAAG (truncated antisense) cut and ligated into *Nsi*I and *Pac*I sites of the expression cassette.

### Cell preparation for microscopy – Confocal microscopy:

encysting trophozoites at four hours post induction were fixed for immunofluorescence microscopy in 3% formaldehyde (all chemicals purchased at Fluka, Buchs, Switzerland unless noted otherwise), permeabilized with 0.2% Triton X-100 and blocked in 2% bovine serum albumine/phosphate-buffered saline (PBS). CWP was detected with a Texas Red-conjugated monoclonal anti-CWP antibody (Waterborne Inc., New Orleans, LA) as a marker for ESVs. Recombinant Bax was detected with a monoclonal antibody (NeoMarkers, Fremont, CA) followed by a secondary Alexa488 conjugated anti-mouse IgG antibody (Invitrogen AG, Basel, Switzerland). An anti-IscS rabbit antiserum (kind gift of Dr. J. Tovar) followed by a secondary Alexa594-conjugated antibody (Invitrogen, Basel, Switzerland) was used to label mitosomes. Confocal microscopy was performed on a Leica SP2 AOBS unit (Leica Microsystems, Wetzlar, Germany).

### Electron microscopy:

Cells were recorded at a magnification of 5'000× using a CM 12 electron microscope (Philips, Netherlands). Encysting trophozoites were prepared for electron microscopy as described previously [14] and were allowed to actively attach to sapphire disks to obtain uniformly oriented parasites for fixation and subsequent sectioning in the dorso-ventral plane. Attached *Giardia* trophozoites were submitted to ultra-rapid freezing prior to fixation to preserve membrane structures optimally.

## Results and Discussion

### *Giardia* mitosomes are not targeted by recombinant Bax

**Figure 1:**
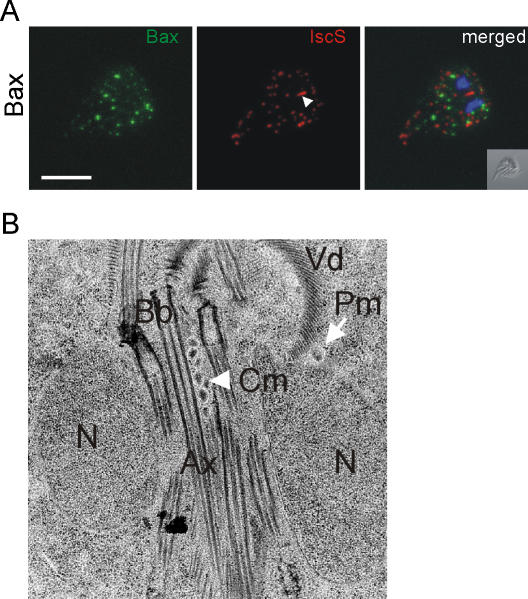
Expression of human Bax in induced *Giardia* trophozoites does not affect mitosomes. A) Confocal microscopy analysis of the subcellular distribution of Bax in transgenic cells. Recombinant Bax (left panel, green) does not localize to mitosomes (middle panel, red) labeled with an anti-IscS rabbit antiserum as a mitosome marker. The signature central mitosome structure is indicated with an arrowhead. The merged images show a clear lack of co-localization. Nuclear DNA is stained with DAPI (blue). Inset: differential interference contrast (DIC) image. Scale bar: 5 µm. B) The central mitosome structure (Cm, arrowhead) is resolved as a tightly packed array of spherical organelles in electron microscopy of adherent cells. The subunits are indistinguishable from individual peripheral mitosomes (Pm, arrow). This organelle structure is unchanged in transgenic trophozoites expressing Bax. N, nucleus; Bb, basal bodies; Ax, axonemes; Vd, ventral disk.

The presence and maintenance of mitosomes in *Giardia* remains a mystery. Specifically, it is unclear whether they still fulfill any essential function or have become effectively obsolete during their considerable evolutionary reduction. We attempted to target and ablate these organelles using the pore-forming Bax protein, and assess the viability of the parasites. This strategy was successful in the evolutionary basal protozoan *Trypanosoma brucei*, which also lacks an apoptotic machinery, and led to release of cytochrome c, mitochondrial fragmentation and cell death [22]. To express the human *BAX* gene in *Giardia*, we engineered a plasmid vector containing the full-length open reading frame under the control of the inducible CWP1 promoter, which is tightly repressed and activated only in trophozoites induced to undergo encystation [27]. The subcellular localization of the recombinant protein was determined using confocal laser-scanning microscopy of transgenic parasites after fixation and permeabilization with detergent. Recombinant Bax was detected with a specific antibody as a punctate pattern in the cytoplasm, reminiscent of peripheral mitosome distribution (Figure 1A, left panel). Surprisingly, double immunofluorescence studies revealed no co-localization with the mitosomal matrix marker protein IscS (Figure 1A, middle panel). Most importantly, Bax did not localize to the mitosome signature structure, the central mitosome (Cm), at the basal body complex in the cell center (arrowhead). This demonstrates that Bax does not interact with these organelles at all, but either forms aggregates in the cytoplasm, or attaches to unknown structures in the cell.

The central mitosome (Cm) is a signature structure, which appears as an elongated organelle between the nuclei in immunofluorescence micrographs, and is faithfully divided and partitioned during cell division [6]. Although the Cm did not seem to be affected by the expression of Bax (Figure 1A), subtle changes might go unnoticed. The morphology of the central mitosome has not been defined by electron microscopy because it is very difficult to obtain a suitably oriented section of the cell center in randomly sectioned cell pellets. To address these questions we made use of a previously applied technique to section uniformly oriented adhering trophozoites in a plane perpendicular to the dorsal-ventral axis [28] for analysis by electron microscopy. With this approach, longitudinal sections through the cell center can be found frequently, which allows a systematic survey of mitosome structures in wild-type *Giardia* and transgenic cells expressing Bax. Figure 1B shows a representative image, which reveals the central mitosome as a tightly packed cluster of small spherical organelles (arrowhead) quite similar to peripheral mitosomes in the cytoplasm (arrow). The clustered organelles are embedded within the basal body – axoneme bundle, consistent with the observed faithful partitioning to daughter cells, since this is among the first cellular structures to segregate in dividing cells [29], [30]. In cells expressing Bax, the morphology of the central mitosome is indistinguishable (data not shown). Together with the absence of Bax targeting to these organelles, this argues against a direct interaction of Bax with mitosomes and suggests a high degree of degeneration during evolutionary reduction. As in *Giardia*, classical caspase-dependent apoptotic pathways and proteins of the Bcl-2 family are absent in other basal eukaryotes such as the protozoan *Trypanosoma brucei*. Nevertheless, trypanosomes and the related *Leishmania* can undergo a form of programmed cell death [31]–[33] in response to external stimuli. In addition, conditional expression of human Bax in *T. brucei* leads to dynamin-dependent fragmentation of the single mitochondrion and release of cytochrome c from the intermembrane space [22], [34]. This indicates that the factors required for targeting Bax to the cytoplasmic side of the mitochondrial membrane and for release of cytochrome c remain conserved in *T. brucei*, even if the rest of the machinery is absent. Programmed cell death has also been postulated for *Giardia*, although direct evidence is lacking and a physiological role is unknown. The fact that Bax is unable to target giardial mitosomes is consistent with the highly diverged status of these organelles.

### Expression of Bax affects cell viability and secretory organelle development

**Figure 2:**
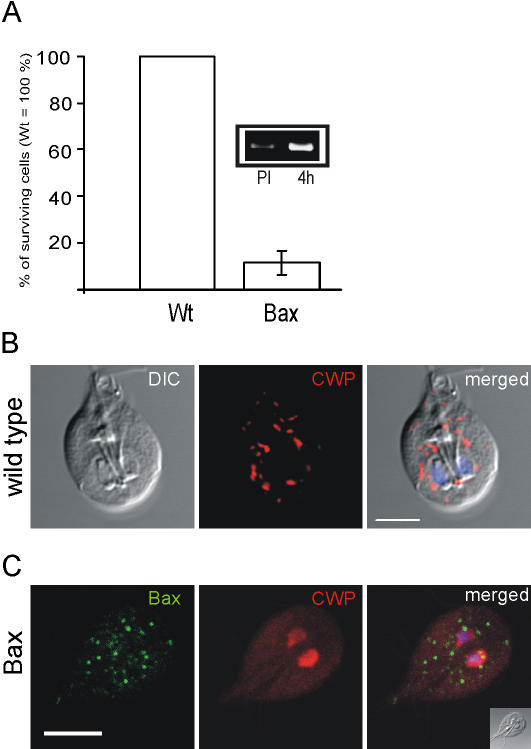
Expression of Bax is lethal for *Giardia.* A) Induced cells expressing Bax stop dividing and die in the course of 6 hours. Inset: semi-quantitative RT-PCR analysis of Bax mRNA levels in transgenic cells pre induction (PI) and 4 hours after induction. B) Subcellular localization of CWPs in ESVs of encysting wild type cells labeled by a monoclonal anti-CWP antibody at 4 h post induction. C) Bax abolishes ESV formation in encysting cells and causes accumulation of CWPs in the cytoplasm and the nucleoplasm. Inset: differential interference contrast (DIC) image. Scale bar: 5 µm.

Despite the fact that Bax expression had no apparent effect on mitosomes, a large proportion of induced transgenic *Giardia* parasites failed to complete differentiation and died. The cells lost motility, the ability to attach to the surface of the culture tubes several hours after induction, and were lysed. Microscopic quantification of this effect at 6 hours post induction revealed that the number of vital transgenic cells was reduced by ∼85% compared to the identically induced wild-type parent strain (Figure 2A). Cells were assessed for morphology, motility and ability to attach to a plastic substrate. This effect was correlated with increased expression of Bax mRNA (Figure 2A, inset).

In induced *Giardia* trophozoites undergoing stage-differentiation, cyst wall proteins are accumulated and mature post translationally in large ESVs before regulated secretion onto the surface of the cells at the final stage of this process. To determine whether death of the transgenic cells was directly linked to encystation, we investigated if formation of ESVs was affected by Bax. To assess encystation in the presence of Bax, we compared ESV formation in wild type and transgenic *Giardia* by confocal immunofluorescence microscopy using an antibody against CWP1, a major ESV cargo protein. The localization and appearance of normal ESVs in wild type cells at 4 h post induction is shown in Figure 2B. At this stage, synthesis into the ER and concentration of cyst wall proteins in growing ESVs is clearly evident in induced cells. Surprisingly, in transgenic cells expressing Bax we found no ESVs. Instead, CWP was detected in the cytosol, and appeared to accumulate also in the nuclei (Figure 2C). The simplest explanation was that ESVs were targeted and compromised by Bax, releasing their cargo in the process. At what stage of development ESVs become susceptible could not be determined. However, the absence of these organelles in Bax-expressing cells, even at early stages of encystation (2–4 h), suggests that emerging ESVs are targeted very early by Bax.

### Deleterious effects on differentiating *Giardia* are linked to conserved functional domains of Bax

**Figure 3:**
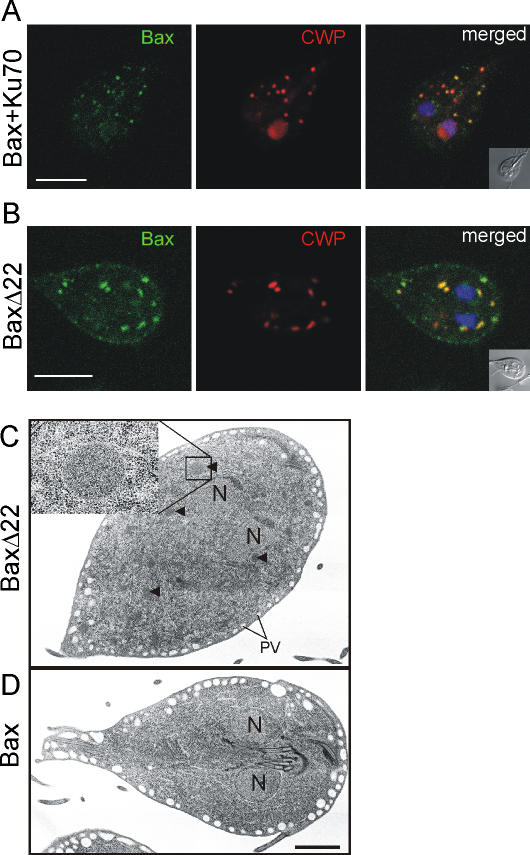
CWP release from ESVs is Bax specific. A) ESVs in encysting Bax-expressing cells treated with the membrane-permeable Bax-inhibiting peptide Ku70 are at least partially protected. Note the co-localization of Bax (green) with (partially intact) ESVs and significantly less cytoplasmic or nucleoplasmic CWP signal than in untreated cells (compare with FIG. 2C).16-May B) Deletion of the Bax C-terminus uncouples Bax targeting to ESV membranes and CWP release. BaxΔ22 lacks the C-terminal 22 amino acids of Bax and localizes to membranes of intact ESV. C, D) Electron micrographs of representative cells expressing Bax or BaxΔ22 at four hours post induction. C) Encysting trophozoite from the population expressing BaxΔ22 showing numerous ESVs with electron dense material (arrowheads). ESV and general compartment morphology are indistinguishable from wild type cells (not shown). Enlarged region shows an individual ESV. Peripheral vesicles underlying the plasma membrane are clearly visible. D) ESVs or organelle remnants are not present in surviving cell expressing Bax. Nuclei, and parts of the microtubule structures of the anterior flagella and significantly enlarged PVs are visible. N, nuclei. Scale bars: 2 µm.

We attempted to determine if the apparent ablation of ESV organelles and concomitant cargo release were due to a Bax-specific mechanism or simply a consequence of over-expression. To test this, we treated transgenic cells with the Bax-inhibiting peptide Ku70 [35] and analyzed the effect on ESV formation after induction. In contrast to the complete ablation of ESVs in untreated controls, organelles containing cyst wall material developed in the presence of the peptide, and leaking of CWPs into the cytoplasm and the nuclei was significantly reduced (Figure 3A). This indicated that ESVs were at least partially protected, suggesting a specific inhibition of Bax analogous to the specific effects of Ku70 in higher eukaryotes. More importantly, the resulting stabilization of ESVs now revealed localization of recombinant Bax to those organelles. This also supports the notion that fast and complete ESV ablation in untreated cells precludes direct observation of Bax targeting. Mitigation of this effect by addition of Ku70 therefore provides evidence for the ability of recombinant Bax to bind to ESV membranes. However, unlike in mammalian cells, the inhibitor peptide does not abolish membrane targeting of Bax [35]. To investigate the specificity of Bax for ESV membranes in more detail we expressed a mutant variant, termed BaxΔ22, which lacks the 22 C-terminal amino acids of the protein which is thought to be involved in pore formation. Cells expressing BaxΔ22 were fully encystation-competent and able to form ESVs. Without its carboxy-terminus, Bax still localized to ESV membranes but no release of CWPs into the cytoplasm was observed (Figure 3B). Taken together, this demonstrated complete uncoupling of membrane targeting and release of cargo in this system, consistent with findings in Bax-expressing yeast and in mammalian cells, demonstrating that organelle targeting of Bax is maintained in the absence of its C-terminus [36], [37]. The latter is still controversial, however, since another study has found that the hydrophobic C-terminus of Bax acted as a mitochondrial-targeting signal [38].

Although the confocal microscopy data suggested that ESV cargo was released into the cytosol as a result of Bax expression, the fate of these organelles remained unclear. To investigate the effects of Bax on ESV compartment integrity in more detail, we performed thin section transmission electron microscopy comparing cells 4 h post induction expressing either Bax or BaxΔ22 (Figure 3). An extensive survey of uniformly oriented and sectioned *Giardia* revealed that >80% of BaxΔ22-expressing cells contained ESVs with an ultrastructure identical to those in wild-type cells (Figure 3C, D) [28]. In contrast, virtually no ESVs were present in cells expressing full length Bax (Figure 3D). In addition, no unusual membrane structures that could be interpreted as “empty” ESVs were observed. The only anomaly was a significant increase of peripheral vesicle (PV) size. PVs constitute a distinctly localized endosomal-lysosomal system in *Giardia*, which is arrayed below the plasma membrane, and involved in endocytic and exocytic processes [39]. Bax-expressing cells contained enlarged PVs (diameter: ∼0.5–1 µm) (Figure 3D), in contrast to encysting wild-type parasites [28] or transgenic cells expressing BaxΔ22 (diameter: ∼100–150 nm). This suggests that PVs may be involved in removing lysed ESVs. Thus, one explanation for the complete absence of ESVs in transgenic Bax expressing cells may be that an autophagy-like process eliminates these compartments after cargo has been lost.

In summary, we show that in the absence of bona fide mitochondria in the early-diverged *Giardia*, conditionally expressed human Bax is targeted to ESVs. Bax alone is sufficient to compromise these organelles, presumably by the same mechanism as for mitochondrial membranes in higher eukaryotes, i.e., pore formation. Consistent with this, we observed release of CWPs to the cytoplasm. Similar to the cytochrome c release from mitochondria, this process depends on the carboxy-terminus of Bax [38] and can be partially inhibited by the Ku70 peptide. Thus, ectopic expression of Bax in encysting *Giardia* results in specific organelle targeting, cargo release, and cell death completely independently of mitochondria.
